# Fashion Companies Pioneering with Eco-Innovations in the Swedish Fashion Industry: Motivations, Resources, and Cooperation

**DOI:** 10.1007/s43615-022-00246-x

**Published:** 2023-01-18

**Authors:** Naomi Le Feber, Martijn J. Smit

**Affiliations:** 1 Utrecht University, Utrecht, The Netherlands; 2 Faculty of Geosciences, Utrecht University, Utrecht, The Netherlands

**Keywords:** Eco-innovation, Circular economy, Fashion industry, Corporate cooperation, Sustainability

## Abstract

Eco-innovations are required to solve the environmental issues in the fashion industry, but their widespread implementation and diffusion is still limited. Both literature and practice indicate that multi-stakeholder cooperation can provide necessary knowledge, scale, and resources that single actors lack. This qualitative research employed an explorative case-study approach to develop an understanding of how and why certain fashion companies in Sweden innovate and what role cooperation has in their eco-innovative activities. The findings suggest the importance of environmental motivations, market opportunities, and cluster effects. These findings contribute to the development of strategies for a sustainable transition of the fashion industry.

**Supplementary Information:**

The online version contains supplementary material available at 10.1007/s43615-022-00246-x.

## Introduction

Sustainability issues and digitalization require extensive environmental innovation in the fashion industry [3, 13]. Environmental innovations (eco-innovations) are all the measures undertaken by relevant actors, such as enterprises, non-profit organizations, politicians, unions and associations, that develop new ideas, behaviors, products, and processes with the aim to contribute to a reduction of environmental burdens or to ecologically specified sustainability targets, and apply or introduce these [47].

Within the fashion industry, the implementation and diffusion of eco-innovations are limited due to the global nature of the industry and the characteristics, practices, and sizes of fashion enterprises [40]. The majority of national fashion industries consist of micro (Rieple et al. 2015; [63], small, and medium enterprises, and these often lack resources, experience, capabilities and knowledge. Consequently, eco-innovative fashion firms rarely operate at the necessary scale to create a change in the fashion industry [15, 24, 28, 33, 36, 51].

Previous studies [13, 15] and fashion industry reports [36] indicate that cooperation with various stakeholders can provide the knowledge, ideas, and resources to overcome these deficiencies to facilitate eco-innovation. Yet empirical research on eco-innovations in the fashion industry has limited its focus to business model innovation [28, 59, 60], value chains [12], and circularity [10]. There is a great diversity of fashion companies that have adopted eco-innovative processes and/or developed such products early on. Yet this diversity of trajectories is not yet reflected in existing empirical research.

We therefore zoom in on the question how different pioneering fashion companies have become eco-innovative, and how this development relates to the regional cluster they are located in. Our contribution to the literature lies in the particular attention to the role of large and small firms, and to the nexus between the firm and its surroundings. It is crucial for policymakers, industry agents, and peers to understand the various trajectories of eco-innovative companies and what these pioneers deem necessary. Understanding how different fashion companies are eco-innovating and whether size or locations matters or not helps with the development of policies and industry practices to support the diversity of fashion companies that are not yet successfully implementing eco-innovations.

We employ an explorative case-study approach to develop an understanding of how and why certain companies bring change in the fashion industry with eco-innovations and what the role of cooperation is in the exchange of knowledge, ideas, and resources regarding sustainability and circularity. Our research is explorative, since the sector is very dynamic, the number of routines is still increasing, and a “shakeout” phase has yet to take place [38]. We focus on the Swedish fashion industry because Sweden targets to lead the global fashion industry in sustainability [37] and is already home to many sustainable companies, both large and small, both young and old.

By means of in-depth interviews, we aim to answer the following research questions:

- RQ1: what is the relationship between the pioneering fashion companies’ size and their ability and willingness to develop and implement (product and/or process) eco-innovations?
- RQ2: why are pioneering fashion companies developing and implementing eco-innovations?
- RQ3: what do pioneering fashion companies perceive as limiting factors to their ability to develop and implement eco-innovations and how do they cope with these?
- RQ4: what are the motivations of individual actors to cooperate *horizontally* or *vertically* in the fashion industry in order to search, develop, and implement eco-innovations?

The paper is structured as follows. After a literature review, we discuss our research methods. We then present our results. In the “Discussion” section, we answer the research questions, and we round off with a conclusion.

## Literature Review

**Fig. 1:**
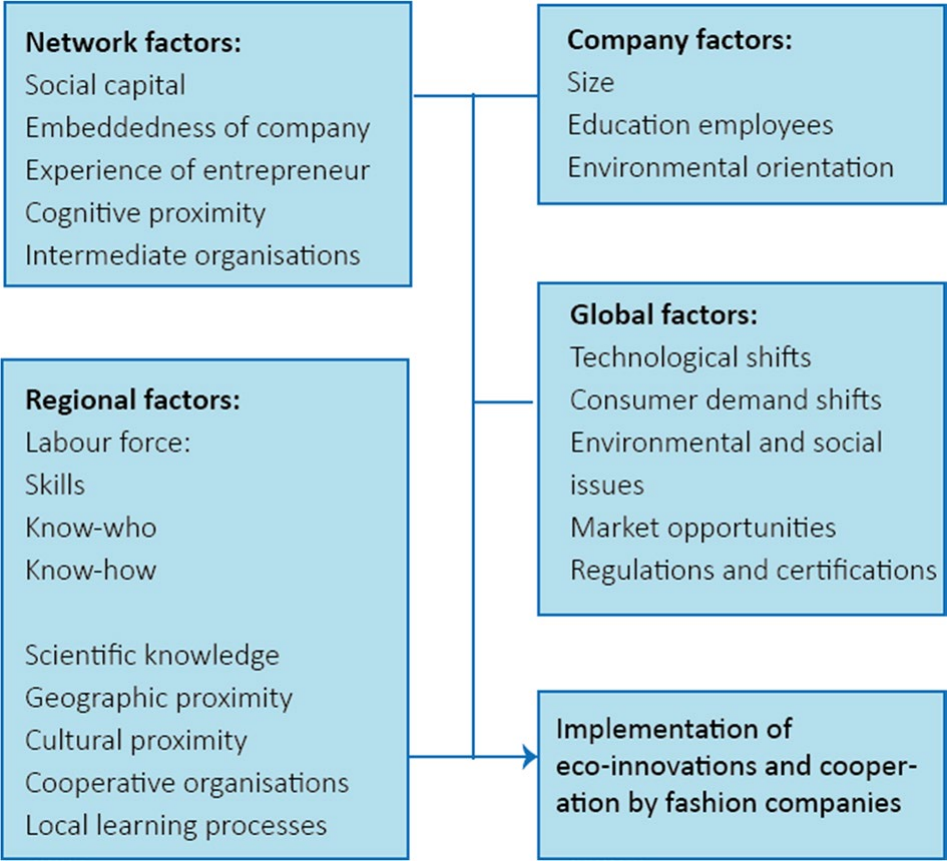
Conceptual model

Recent years have seen the rise of eco-innovations in the fashion industry [28, 57, 59, 60]. Since an industry-wide solution to environmental problems is still lacking [13, 28], it is important to understand how an industry changes systematically. Individual companies make choices to innovate but do so based on a metaphorical “landscape,” an environment of social and technological developments, as Geels and Kemp [18] call it. In our conceptual model (Fig. 1) we classify these as global factors. Moreover, they do so in an actual spatial setting, which we represent as regional factors. Network factors cover effects that are sometimes global and aspatial but often very localized [54].

We will explore the innovation process at the firm level first and then briefly explore how clusters drive innovations and develop into innovation systems, how this is related to the fashion industry and environmental innovations, and what drives the environmental innovativeness of companies and entrepreneurs.

### Eco-Innovations and Business Model Innovation in the Fashion Industry

Companies and entrepreneurs are allocated an important role in sustainable development, both by academics and policymakers. Companies and entrepreneurs that seek to induce environmental, social, and ethical change are identified as agents of change [2, 30, 65]. The literature labels them as green companies or entrepreneurs [2, 14, 25, 65]. In the fashion industry SMEs lead the sustainable transition [15]. Larger enterprises then follow with large-scope sustainable entrepreneurial initiatives, thus taking the sustainable transformation of an industry to the next level, because only they can make “significant structural changes” [10], p. 7). New entrants, SMEs, and large enterprises have complementary skills and challenges regarding sustainable entrepreneurship,therefore, a co-evolution is more likely to result in sustainability than the actions of either one the two alone [25].

The increase of fashion companies implementing various environmental innovations, ranging from low-risk incremental innovations to high-risk innovations re-architecting products and systems, often requires different business model innovations [59, 60]. This process of change is driven by socioeconomic and cultural macrotrends such as changing consumer demands and technological innovation [3, 36, 40].

Eco-innovations can be technological, organizational, social, or institutional in nature [47]. Among technological innovations, a distinction is usually made between environmental product innovation and process innovation [33, 47]. However, technological and social shifts alone cannot deliver change. Business model innovation (BMI) is expected to yield higher returns than product or process innovations [40]. Sustainable business models might additionally be more resilient and provide diversification and value cocreation opportunities [19]. However, the literature on sustainable business models is recent, and the literature is fragmented. As a result, the approaches and their definitions, boundaries, and characteristics are still unclear. Moreover, distinctions or synergies between circular and sustainable business model innovations are not yet clear [42, 60]. While researchers are focusing on understanding and describing sustainability and circularity in business models, businesses are already being pushed to transform their business model by circular or sustainability thinking, also in the fashion industry [15, 42], and even in declining clusters [9].

The concept of business models emerged in the 1970s in information technology. It gained popularity in the 1990s, with the e-commerce boom when new and innovative revenue mechanisms were introduced. Recently, business model innovation increasingly receives attention in specific areas such as sustainability, circularity, and technology [10, 19, 42]. Since a business model is a concept only, it requires translation into concrete activities such as business structure (departments, human resources), business processes (workflows, responsibilities), and infrastructures. Business models are subject to external pressure and inherently subject to change. When a company decides to adopt a new business model or to change an existing one, capturing and visualizing this model will improve planning, change, and implementation [39].

Today, sustainable business models, among which circular business models, are increasingly a source of competitive advantage [19]. The various definitions of sustainable business models in the literature have in common that they are a modification of the conventional business model concept, either integrate incorporating sustainable concepts, principles, or goals, or integrate sustainability into their value proposition, value creation, and delivery activities. A sustainable business model thus incorporates pro-active multi-stakeholder management as well as monetary and non-monetary value creation, in which the non-monetary value can be understood as absolute or relative reductions of negative environmental and/or socioeconomic effects. It thus offers a long-term perspective [19, 42].

Business model innovation can occur in different ways, and its dynamic and continuous process distinguishes it from “single initiative” product and process innovations [42]. It can involve “the development of entirely new business models, the diversification into additional business models, the acquisition of new business models, or the transformation from one business model to another” [19]. Transformations are particularly interesting: these can encompass the business model as a whole but also some of its constituent parts, where a smaller or larger process innovation can be said to occur in case it creates “significant positive and/or significantly reduced negative impacts for the environment and/or society, through changes in the way the organization and its value-network create, deliver value and capture value (i.e., create economic value) or change their value propositions” [6].

Many studies do not contextualize the stage or form of business model innovation they are contributing to, possibly contributing to a design implementation gap. Moreover, the incorporation of human behavior aspects such as leadership and organizational culture in enabling transformation towards a sustainability-oriented business model is limited [42].

### Regional Clusters as Drivers of Innovation

Actors, networks, and resources tend to cluster in regions, and this concentration drives regional specializations and entrepreneurial activity into certain technologies and industries [8]. This also holds for fashion companies, which have traditionally clustered in core areas of major cities, and there they increasingly focus on design and knowledge-intensive activities. Labor-intensive production has, on the other hand, been outsourced to suppliers and subcontractors in low-wage countries. Therefore, the fashion industry nowadays is a global industry with design and R&D activities clustered in high-wage areas and production and assembly clustered at low-wage areas [22, 52, 48].

The same urban clustering process holds for a newer generation of technology-driven companies in the fashion industry [36, 57]. These strive to use fewer resources and belong to what some call “green-tech.” Their clustering in large urban areas is seen as a strategy to support innovative production and R&D activities. Whether their clustering actually generates tangible advantages is not known to many firms, but their simple perception of having to join a cluster will also convince them to colocate [61], while knowledge exchange can occur just as easily through informal routes as it can through purposeful cooperation [46].

Actors, networks, and resources tend to cluster into regions, and this concentration drives regional specializations and entrepreneurial activity into certain technologies and industries [8]. These clusters are places that stimulate collective learning and continuous innovation through inter-firm communication and knowledge transfer, socio-cultural structures, and an institutional environment [4, 22, 35, 43]. Exchange processes are enabled by technological proximity or a common value chain between actors in the cluster. Furthermore, enterprises in a regional cluster exploit both local, place-specific resources as well as external, global knowledge for their innovation activities [4]. In particular, export-oriented clusters are more likely to have national and international linkages [44, 55].

Local resources include unique combinations of knowledge and skills by the labor force, the presence of several specialized suppliers, local learning processes, technology transfer, and spill-over effects supported by geographical and cultural proximity, and cooperative organisations. Localized knowledge can be formal scientific knowledge, and informal knowledge about know-how, knowledge and skills in specific technologies, and know-who, information about persons with special knowledge [4]. However, in reality, actors may not cooperate beyond what is in their individual interest in a fragmentized and competitive environment. More important is the size of the agglomeration, the number and natural selection of companies that can benefit from the available opportunities [55].

Both clusters and regions are geographical manifestations of a territory, which can be viewed as a system of relationships rather than a geographical entity. It is built and shaped by all its actors. The territory can also be a place of trust [13, 53]. Local cultural production activities are associated with social relationships, which together create patterns of entrepreneurship and innovation [53]. In creative clusters, trust is important to facilitate interaction and knowledge exchange. Especially for small and medium-sized enterprises (SMEs), the territory serves as a source of resources and competences which are hard to acquire internally. The internal production of knowledge and capacity appears to be limited by diseconomies of scale [13]. Since the majority of the fashion industry consists of SMEs that individually lack the reach to create industry-wide change [15], the territory is of crucial importance for environmental innovation in the fashion industry.

### The Importance of Building Connections

Building connections with a diversity of stakeholders and enhancing their involvement in the territory might be the best feasible solution to implement a range of sustainable innovations and strategies both at the industry level and enterprise level to develop a sustainable industry [13]. The efficient implementation and scalability of eco-innovations are limited due to lack of knowledge, competitiveness, and resources among eco-innovative fashion companies and uncertainties in the supply causing actors to hold on to unsustainable, traditional techniques and practices (European Commission 2019,Hermanson et al. 2018b; [28, 33, 36, 40],Sandvik and Stubbs 2019a; [62]. As a response to uncertainty and risk, fashion companies develop connections. They need to acquire decisive information by quickly accessing external information sources and capture the right trends in order to ensure competitive advantage [58]. Eco-innovations are increasingly leading to a competitive advantage [62].

Little is known on the exact connections needed to foster innovation and its adoption in the fashion industry, but we do know that *social capital* is an important contributor to the development of networks and that the embeddedness of a company matters. The key concept of social capital covers the social relations embedded in clusters, including norms, values, customs [50], and mutual trust (Hermanson et al. 2018b). Yet such local or regional networks do not exist in isolation. External linkages also contribute to regional prosperity by providing access to valuable resources such as knowledge and market opportunities [50]. Focal companies foster learning among network actors by transfer of knowledge embedded in products or processes or by teaching processes which they acquired through external interactions [20].

Finally, the fashion industry is a *creative* industry characterized by symbolic value and knowledge. This kind of knowledge involves highly complex and dynamic actor-centered practices based on tacit knowledge and a common understanding of know-how and know-who. Learning and innovation processes happen through doing, using, and interacting [23].

The cognitive proximity between actors in a local cluster facilitates the acquisition of new knowledge and the spread of innovation [1, 7]. However, when a cognitive distance exists between actors, knowledge brokers are necessary for the transfer and exchange of knowledge [23, 29]. Hence, cooperative intermediate organizations can facilitate successful technological adoption and commercialization across innovative networks, for example, by supporting R&D between research, industry, and government actors [29] or by transfer of technology and commercialization of academic-generated knowledge [23]. This indicates the importance of the presence of intermediate organizations for collaboration and the implementation of eco-innovations in the fashion industry, which requires a diversity of actors from research, industry, and government.

## Methods

We employed an explorative case-study approach with information-oriented case selection to understand why and how pioneers are bringing change in the fashion industry with eco-innovations. The Swedish fashion industry was selected as a relevant industrial context to research the implementation of eco-innovation based on previous studies on the Swedish fashion industry [10, 22, 41, 56] and the presence of research institutes and projects, companies, and business organizations solving the fashion industry’s environmental issues through innovation, research, cooperation, and education. Furthermore, Sweden aims to lead the transition to a sustainable and circular economy of the textile and fashion sectors through the national platform Textile & Fashion 2030 (Nejderås et al. 2018).

### Case Selection

The objective of the research was to acquire the greatest diversity of information and perspectives. We therefore selected critical cases with maximum variation (Flyvbjerg. Maximum variation between cases provides insight into fashion companies’ reasons for eco-innovations, perceived limitations and coping mechanisms, and lastly cooperative behavior to facilitate eco-innovations which might be different depending on size, geographical location, the niche market they operate in, and/or the type of innovation(s) they implement. This has the added benefit of maximizing the usefulness of the acquired information from our relatively small sample. All selected companies take a leading position in the transition to a circular economy in the Swedish fashion industry as a whole or within their niche market. For the expert interviews, a variety of experts from different types of organizations has been approached. Included in the research are the perspectives of industry, governmental, non-governmental, and research and education organizations.

The selection of critical cases involved analysis of companies’ and organizations’ websites, sustainability reports and stated sustainability measures, and participation in current national initiatives: Mistra Future Fashion, Textile & Fashion 2030, and STICA. In total, nine fashion companies were interviewed. Moreover, six critical experts from different types of organisations were interviewed. Their selection was based on their involvement in the sustainable and/or circular transition of the Swedish fashion industry to gain an insight into industry-wide perceived reasons for developing and implementing eco-innovations, the perceived limitations and different cooperative relationships to enable eco-innovations in the Swedish fashion industry. An overview of the companies and experts’ affiliations can be found in Appendix.

### Data Collection and Data Analysis

The interviews have been conducted between October 2019 and January 2020 through phone call or face-to-face and have varying lengths between 40 and 90 min. The interviews were semi-structured and guided by a topic list. The interviews were recorded with permission, fully transcribed, and then analyzed with the Nvivo 12 software. Two phases of coding were carried out: firstly, open coding, and secondly, axial coding.

## Results

Fashion companies pioneering with eco-innovation may be different in terms of size and product niche (as shown in Table 1), yet they also have many similarities. Of the nine interviewed companies, no fewer than seven have integrated environmental innovations and commitments in the business model at the time they founded and built their brand. These companies are also relatively young, established during the last 20 years, compared to just one long established company.

**Table 1:** Eco-innovations implemented by interviewed fashion companies

Implemented innovation		Company
**Product**	**Exchanging product component** Sustainable materials, fabrics, packaging, yarns	AWAN No Mans Label Swedish stockings BOOB Dedicated Röhnisch Nudie Jeans Arket
	**Integration new product component** Transparency of supply chain/production processes	Dedicated Nudie Jeans Arket
	**Optimization product component** Durability, prolonging life by repair/reuse	AWAN Nudie Jeans Arket
	**Reclaiming and recycling** Of own products	Swedish Stockings Nudie Jeans
**Process**	**Input raw materials** Those who source sustainable raw material/yarn for fabrics themselves	AWAN No Mans Label Arket
	**Production process** Automation, on demand, 3D sketching, 3D knitting, digitalisation of fabrics	AWAN T-Studio No Mans Label Swedish Stockings Nudie Jeans

For most of the entrepreneurs, environmental motivations are the reason for setting up their brands. They have a background in the fashion industry and encountered its environmental, economic, and/or social issues. Often mentioned were pollution, terrible working conditions, wastefulness, and overproduction caused by the contemporary linear, fast fashion system. The entrepreneurs saw a business opportunity in solving these issues with certain innovations. All of them understand the social, economic, and environmental value and long-term profitability of a sustainable or circular business.

A sustainable business and corresponding eco-innovations require specific knowledge. This kind of knowledge is internalized by sustainable pioneers in the Swedish fashion industry. Most of the interviewees emphasized the importance of knowing their whole supply and value chain for sustainable design, production and logistics, for reducing emissions, water use and chemicals, and for enabling transparency and recycling. This requires knowledge of the production process and the products. Furthermore, on-site experience at factories helps to see what production processes are involved and which are necessary for sustainable or circular production.

Circularity requires knowledge of how to reuse material and products for consumer purposes. Furthermore, companies need to know and understand their value in a circular economy, how they can be a partner to another company, and which companies they need. With this specific knowledge, the pioneering companies have been able to implement a wide range of eco-innovations. Table 1 schematically shows the innovations implemented.

The most prevalent kind of product innovation is exchanging a product component, which is, in the fashion industry context, often yarns, fabrics, zippers, buttons, or the packaging of the final garment. Most of the companies have implemented this since their foundation. Hereafter, they have continued implementing other innovations relevant to their business model. The longer established company differs slightly from the others, because its implementation of this innovation is more gradual.

The results indicate that companies that have integrated environmental value and goals in their business model and strategies move faster with the implementation of eco-innovations. Some companies can be considered as pioneers in eco-innovation, because they lead the industry in terms of production chain transparency, technology, reuse, recycling, and/or repairing programs or by being the first to offer a type of sustainable garment.

Yet almost all the companies have mentioned factors that have hindered or currently limit their ability to implement certain environmental innovations. Some factors are shared by many; some are only affecting a few of them. Nonetheless, they actively look for ways to solve or circumvent the limiting factors they mentioned. The two most encountered limiting factors are lack of demand and lack of qualified personnel. Too little demand for eco-innovative products disables companies to meet the large volumes requested by their suppliers. Consequently, they have restricted capacity and leverage on their suppliers to demand environmental product and process innovations.

Furthermore, some companies, notably the smaller ones, lack people with certain skills or knowledge, which are necessary for implementing more complex innovations or for implementing multiple innovations simultaneously. Consequently, companies need to prioritize what to innovate in order to keep their business profitable. This makes eco-innovation a timely process.

Nonetheless, they actively look for ways to solve these limiting factors. Although some companies lack specific knowledge of certain innovations, they have strategies to acquire the necessary knowledge. One of these strategies is formal education, for example, about sustainable materials, circular design, or working with datasets and coding. Another strategy is informal: keeping a curious mindset, being eager to learn and to stay up to date. Lastly, the eco-innovative companies all have an internal culture of freely sharing information, ideas, and experiences among colleagues, different departments, employees, and management. This enables everyone within these companies to know and work with sustainability. In this aspect, the pioneers differ from other Swedish fashion companies. General observations of Stockholm Fashion District, Boras Ink, Smart Textiles, and Textile and Fashion 2030 indicate that many companies lack knowledge of their business model, their supply chain, and how to work sustainable or circular.

### Reasons Why Interviewed Fashion Companies Eco-Innovate

The interviewed fashion companies have multiple reasons to implement eco-innovations. The interviewees perceive certain developments as factors pushing them away from the current narrative in the fashion industry, which is linear, toward a more sustainable fashion industry. Pushing factors are reasons why the interviewees perceive they *have to* implement eco-innovations, because it is necessary. These are environmental challenges, regulation and legislation, overproduction, and transparency. Pushing factors differ from the reasons that make eco-innovations *attractive* to them, which are labelled pulling factors. These are consumer demand and awareness, market and technological opportunities, and competition. We will discuss these below.

### Pushing Factors

Environmental challenges have pushed several entrepreneurs to start a new business, and they keep pushing them to make more sustainable design and production decisions. The pioneers are committed to lower their negative impact on the planet to the minimum [49]. A rising awareness of these challenges is noticeable in the whole Swedish fashion industry. Especially, Boras Ink sees an increase in applications for start-ups with a reuse or resell business idea as an alternative to producing new fashion.

Furthermore, eco-innovations can be pushed by formal legislation and regulation (cf. [45], and by informal self-imposed regulations by fashion companies (cf. [64]. The results show that all the eco-innovative companies have formalized their sustainability work with public strategies, commitments and goals, and codes of conduct. Some have concrete goals to switch completely to sustainably sourced and recycled materials in the nearby future. Others already produce exclusively with sustainable materials and therefore have different commitments and goals. These interviewees publish yearly sustainability reports, audit and monitor all their suppliers, and work toward circularity and transparency of their supply chain.

Overproduction and transparency were only mentioned by a few respondents whose competitive asset is built around offering a solution to these issues. Therefore, these are considerably less strong push factors than environmental challenges and legislation and regulation which were experienced by almost all.

### Pull Factors

The most important pull factor that was mentioned by all the companies is consumer demand and awareness of environmental issues in the fashion industry. Consumers are increasingly aware of the environmental, social, and overproduction issues related to their clothes [34]. They demand good quality and durable and sustainably produced clothing. All the companies think it is important to consider what consumers want. Moreover, some pioneers actively try to influence consumer demand, steering it toward their products. They do so by creating awareness about environmental issues and change consumer perceptions about sustainable options.

The second most important pull factor mentioned by almost all the respondents is market opportunity. Where in the previous factor the demand was already there, in this case, the companies noticed a gap in the market and see a possible demand for certain eco-innovative products. Innovations need to deliver a solution to a consumers’ problem, otherwise there is no market for the innovation. Lastly, most pioneers understand the technological opportunities that can enable sustainable and circular design and production. Technologies such as blockchain technology [21], automated 3D production, and data structures make eco-innovations attractive and accessible for entrepreneurs. Some start-ups in the fashion industry are increasingly data-driven companies.

### External Inspiration and Information Sources and Cooperative Relations

Different external sources and relations are experienced by pioneers as guiding and supporting to their eco-innovative journey, even though most of their ideas originate from within and they possess most of the needed knowledge and skills for elaborating these ideas. Their stories revealed that some external relations are created purposefully. A distinction can be made between relations that serve as a source of inspiration or knowledge and those that helped the companies in setting up their sustainable business or production. The most important and most turned to inspiration and information sources are other fashion companies, suppliers, sustainability and fashion reports, and research and educational institutions, programs, and projects.

Firstly, knowing what other companies are doing is mentioned by six out of eight companies as an important way to gain a competitive advantage in offering sustainable options, being transparent, and finding out fast about newest materials and products. Furthermore, they think sustainability is co-created with other companies. They share their experiences with other companies directly or within intra-regional cooperative business organizations. Stockholm Fashion District, Boras Science Park, and Textile and Fashion 2030 are organizations with a physical location where companies and a variety of actors (academia, industry and society actors, and public organizations) can gather, connect, and cooperate at lectures, projects, workshops, and seminars. These organizations inform companies about sustainability and circularity and how eco-innovations can be implemented by working together with other actors.

Secondly, suppliers provide information on currently viable, accessible, and new sustainably sourced and produced fabrics and garments. Thirdly, reports and articles provide information about sustainable innovations and developments which are happening now or will be viable in the future.

Fourthly, the interviewed pioneers are characterized by their interest in sustainability and innovation research, interaction with research and educational institutions and researchers, and participation in research programs and projects. In particular, they want to know of the latest research on sustainable materials, technologies, products, and production processes. Some pioneers are participating in research projects or programs because they value being part of the conversation and having access to the perspectives of all different actors involved in eco-innovation.

Start-up pioneers located at Borås University value access to lectures and proximity to other entrepreneurs and researchers, thereby providing them new ideas and information on new developments. Pioneering companies also cooperate with organizations, suppliers, and other fashion companies at an extra-regional level to improve social and environmental conditions in their supply chain and the local communities and nature that their businesses affect. Cooperative relationships are not so much a source of inspiration but more of a source of information and a means to achieve their social and environmental goals. Firstly, a few pioneers (Dedicated, Nudie Jeans, BOOB) cooperate with organizations because they are committed to combat social and environmental issues in the whole fashion industry. Organizations can have a wider impact than they as a business have alone. They support organizations that work for what they believe in and are connected to their supply chain or business.

Secondly, the companies build strong relationships with their suppliers. It is important that their suppliers meet criteria for sustainable production, working conditions, and/or work with sustainable materials. Some only work with fair trade and GOTS-certified suppliers (Nudie Jeans, BOOB, Dedicated). Lastly, four interviewed fashion companies cooperate with other brands. They think sharing experiences and knowledge about sustainability helps to move the industry forward. Swedish Stockings sees the benefits of cooperating with competing hosiery brands because they want the same thing out of it. However, they worry about the issue of splitting the results equally.

Nudie Jeans shows that this is possible when you are transparent about the suppliers you work with. Then, transparency can enable inter-firm cooperation between fashion companies sharing suppliers. This is especially useful for small brands that do not have leverage at their suppliers to push for change. Nudie Jeans cooperates with other brands to share audit and training costs, request fair pay and working conditions, and push the use of sustainable energy sources and water management systems.

## Discussion

Our analysis was performed in late 2019 and early 2020, just before the Covid-19 pandemic. Given both the general slowness of sustainability transitions [31] as well as the mixed effects of previous crises [17], we believe our results stand unchanged after the pandemic. On the other hand, the particular sample of cases, based on information richness and maximum variation in size, geographic location and niche, asks for caution when generalizing our findings across the fashion industry as a whole.^1^ The participating cases are all pioneering with innovations for a sustainable or circular economy, or actively involved in improving the sustainability and innovativeness of Swedish fashion companies. Seven of the nine company cases have been in business for several to 20 years, proving the viability of their sustainable business and eco-innovations.

### RQ1: Different Roles for Companies Differing in Size?

The relationship between company size and the development and implementation of eco-innovations is disputed in previous studies. SMEs are identified as leaders in the sustainable transition of the fashion industry because they make up the majority in numbers. However, they are found to lack the reach and resources necessary to create industry-wide change [11],European Commission 2019; [58]. General research on the drivers of eco-innovation indicates that larger companies are more likely to implement eco-innovations and that the extent of these innovations is also larger [26, 32].

In contrast, Caniato et al. [11] as well as Hockerts and Wüstenhagen [25] indicate that start-ups and SMEs have a disruptive role in the early stages of an industry’s transformation toward sustainability by implementing more radical innovations, while larger companies follow with incremental and large-scale innovations. Previous case study research on the development and implementation of eco-innovations in the fashion industry either studied micro or small and medium companies [5, 9, 28],Rieple et al. 2015) or large companies [33, 56].

We found no evidence that the size of companies matters for their ability or willingness to develop and implement eco-innovations, or in the kind and impact of their eco-innovations. In fact, we confirm small companies are often able to identify and tackle specific problems better than their large counterparts [28].

### RQ2: Driven by Environmental Motivations, Guided by Market Opportunities

Regardless of size, all the participating companies identified a market opportunity in solving environmental and/or social issues. These market opportunities are created by increasing consumer awareness of environmental issues, thereby increasing demand for sustainable fashion. The findings suggest that environmental motivations and identification of market opportunities for eco-innovative businesses, technologies, products, and processes might be more important than company size in explaining and driving the sustainable transition of the fashion industry.

The companies of this study are pushed to develop and implement eco-innovations by an intrinsic motivation to solve environmental challenges. They publicly monitor their sustainability with informal self-imposed regulations such as public sustainability strategies, commitments, and goals. Through this organizational innovation they show willingness and means to continually innovate. The cases also show that even the smallest companies are able to produce in a sustainable and/or circular way, stay profitably, and grow. The environmental motivation is shared by everyone in the company and motivates employees to gather the necessary knowledge to enable eco-innovation. These companies place environmental value at the core of their business, which Pal and Gander [40] describe as a strong approach to business model innovation. Clearly defined rules also prevent companies from delaying measures or opting for low-cost innovation, and allow them to continue operating in a linear way [56]. Furthermore, this finding corresponds to Hussain’s [28] findings that companies creating and capturing value through product and process innovation are more competitive than those that only replace materials.

### RQ3: Clustering Supports Diffusion of Eco-Innovations

Regional clusters are still the dominant places that stimulate collective learning and innovation through localized networks of innovators and cross-fertilization between research institutions [4, 23, 35, 43]. The Swedish fashion industry is no different, and it is clustered in Stockholm, Gothenburg, and Borås by order of magnitude [22].

However, we find that only in Borås the local clustering of fashion companies, academia, research institutes, and organizations is driving innovation in fashion and textiles in the way the literature identifies. All the interviewed companies from Borås were start-ups developing and implementing a certain eco-innovation. These start-ups acquired inspiration and information on new innovations from local academia and research institutes. Furthermore, the development and implementation of their eco-innovation are aided and partially facilitated by organizations supporting innovation and business development.

In the Swedish fashion industry, specific co-innovation projects act as gatekeepers for the network as a whole by integrating internal and external knowledge of eco-innovations. This external knowledge is often acquired through cooperation with international organizations and suppliers that enable sustainable manufacturing. They share this knowledge in cooperative organizations with other companies such as STICA, or with companies with which they share suppliers.

Temporary clusters might play a more important role than co-location in driving the widespread development and implementation of eco-innovations in the Swedish fashion industry. The Stockholm Fashion District and the Boras Science Park are organizations organizing events, workshops, and lectures where a variety of actors from the Swedish fashion industry, research institutes, public organizations, and society gather, connect, and share information and experiences about sustainability in fashion. These findings confirm the fundamental assumption in the literature that spatial proximity enhances knowledge transfer to companies with poor cognitive abilities and strengthens their absorptive capacities [13]. Furthermore, they align with Hauge et al. [23] who found that learning and innovation processes in the fashion industry happen through doing, using, and interacting, because knowledge is complex and tacit. Therefore, it requires a common understanding of know-how and know-who. The interviews with companies suggest that they lead in the sustainable transition because they possess this understanding of know-how and know-who.

### RQ4: The Importance of Industry-Wide Cooperation

Fashion companies cannot achieve full sustainability and/or circularity on their own. Their supply chains are global because manufacturing processes are spread across the world. This reinforces the importance of industry-wide collaboration, since all the actors in the supply chain are dependent on each other (Sandvik and Stubbs, 2019b). This understanding is shared by the companies and organizations participating in this study. This means that physical proximity alone is not sufficient for the widespread development and implementation of eco-innovations. Attention should also be paid to social relationships, cognitive proximity, and intermediate organizations, as suggested by the literature [7, 13, 23].

Since Swedish fashion companies operate on a global level, their networks and innovation processes are not locally or regionally bound. It is therefore useful to apply the concept of the territory when studying innovations in the fashion industry. The fashion industry can be viewed as a system of relationships, which provide resources and competences that are hard to acquire internally because of diseconomies of scale [13], p. 15,European Commission 2019).

The interviewed eco-innovative fashion companies apparently operate in a different system of relationships than the majority of companies that are not yet eco-innovative. They have purposefully established relationships within their supply chain and with research partners to develop and implement innovations. However, most of them have no direct interaction with other eco-innovative companies and organizations that stimulate and facilitate sustainability in the Swedish fashion industry except for interviewed start-ups in Borås. The increasing number of companies approaching organizations, such as Textile and Fashion and the Stockholm Fashion District, suggests that the majority of fashion companies in the Swedish fashion industry understand the importance of becoming sustainable but still lack this particular knowledge. These organizations therefore provide them with the information and tools to fashion companies that do not know how to become sustainable and where to start this process, acting as knowledge brokers to spread environmental practices and innovations across the fashion industry [23, 29].

However, we found the interviewed innovative fashion companies already possess knowledge of know-how and know-who. For them, lack of such knowledge is not a motivation to increase cooperation (cf. SQ3). Here, our findings contrast those of Pedersen et al. [41] who claim large companies are better equipped toward sustainability transitions. Our findings are supported by the innovations they implement across their supply chains and in their participation in research projects and programs. The interviewed companies know their supply chains and how their products are produced. Furthermore, they are aware of prominent research findings, ongoing projects, and programs which are useful for reaching their sustainability goals. As argued above, this might be explained by their environmental motivations and scanning of market opportunities in sustainable fashion. Therefore, they do not rely upon other actors for resources and knowledge to become more sustainable. Rather, their connections are constructed as a means to enable them to continually innovate, select, and cooperate with suppliers, academics, companies, and organizations to develop and implement eco-innovations in the whole supply chain while also contributing to industry-wide systematic change. This finding validates the recommendation provided by De Chiara [13] for the shift toward a sustainable fashion industry.

A large gap still exists between the pioneering fashion companies and the majority of Swedish fashion companies in terms of knowledge, strategies, and measures taken to become more sustainable and innovative. However, the rise of organizations that facilitate and stimulate a sustainable fashion industry transition suggests that the transition process is scaling up. The direction of transition processes is usually directed and influenced by certain influential actors, either positively or negatively [65]. Influential sustainable companies can challenge the dominant mode of production and consumption of a regime by means of market, discursive, and political impact [27]. The findings indicate that organizations and researchers can also play a key role in challenging the existing regime of the fashion industry by protecting and exposing eco-innovative companies, in the literature referred to as niches [65].

Further research is needed on how eco-innovative companies, researchers, and organizations can influence policymaking at different levels and how they influence the development and implementation of sustainable practices and strategies across the whole fashion industry. A suitable approach would be a longitudinal study encompassing all actors in the fashion industry’s supply chain, allowing for analyzing the rise of certain influential actors and evolution of industry-wide relationships. This would enable an evaluation of how the fashion industry at the national, regional, and global levels transitions over time. Moreover, it would allow for analyzing the pace at which innovations occur. Therefore, it would contribute to deeper understanding of how the global fashion industry can transition from a linear to a circular regime.

## Conclusion

The sustainable transition of the fashion industry is driven by innovative companies of all sizes that identified market opportunities in solving environmental issues. Compliance to self-imposed regulations and the willingness to create and capture value through continued product and process innovations explain why the interviewed companies are at the front of the transition and are fast and competitive innovators. For fashion companies that aspire eco-innovations, the results suggest the importance of identifying a market opportunity in solving an environmental issue, whether that is a process or product innovation, that is attainable for them and which they find important to address.

In order to understand how the sustainable transition of the fashion industry can be scaled up to bridge the gap between eco-innovative and motivated companies and the majority of non-eco-innovative companies, the Swedish fashion industry proved to be a valuable case study. The study findings indicate that local clustering of fashion companies, academia, research institutes, and organisations can support the dissemination of knowledge and practices to enable the development and implementation of eco-innovations. However, suppliers and subcontractors responsible for all the production activities where most eco-innovations have to be implemented are not co-located with fashion companies.

Therefore, temporary clustering of different actors from the supply chain rather than co-location might set into motion an industry-wide shared understanding and interest for the development and implementation of eco-innovations. In the Swedish case, different organizations with physical meeting and testing locations proved to be important initiators for the temporary clustering of different actors. These organizations were able to kick-start the dissemination of sustainable and circular knowledge and practices, and to connect actors across the supply chain as a whole. The results suggest that these physical meeting and testing locations for different eco-innovations might be helpful for fashion companies that are searching for eco-innovative (market) opportunities they can grasp. In further research, it is necessary to validate whether the clustering we described actively drives eco-innovation and in what ways. For example, it might be valuable to discern the role of the local network in eco-innovation processes, which depends on the different roles of local actors [20].

### Electronic supplementary material

Below is the link to the electronic supplementary material.

Supplementary file1 (PDF 859 KB)

## Data Availability

Materials from the interviews underlying the present work cannot be made available for wider use.

## Appendix A.1

Table 2

**Table 2:** Approached fashion companies

Size	Company	Location	Product:	Process:
**Micro (1–9)**	As We Are Now	Stockholm	X	X
	T-studio	Borås		X
	No Mans Label	Borås	X	X
**Small** **(10–49)**	Boob Design	Stockholm	X	
	Swedish Stockings	Stockholm	X	X
	Dedicated	Stockholm	X	
**Medium** **(49–249)**	Röhnisch	Stockholm	X	
	Nudie Jeans	Göteburg	X	X
**Large** **(250 +)**	Arket	Stockholm	X	X

## Appendix Table A.2

Table 3

**Table 3:** Interviewed experts

Type of organization	Name and location	Involved parties and financiers	Activity	Interviewed
Industry	Stockholm Fashion District	Association of Trade Partners Sweden	Business platform for fashion in Sweden	Head of public relations
Government	Textile & Fashion 2030—Borås	Smart Textiles; The Swedish school of Textiles; Swedish Fashion Council; RISE; Teko; Svensk Handel	In collaboration with business and academia encouraging and coordinating education, research, innovation with the aim of supporting environmentally friendly development in fashion and textiles	Project manager
Research	Smart Textiles Borås	Science Park Borås; RISE; Swerea IVF; Incubator Borås; Vinnova, Västra Götaland region; Sjuhärads Kommunalförbund	Engine of the Swedish textile industry since 2006 with over 500 research and business projects. It is an innovation environment in northern Europe for the fashion and textiles and textile technology	Innovation leader
				Associate professor/Responsible Smart Textile Technology Lab
Incubator	Borås Ink	Borås Stadshus AB	Personalised and customised business development for technology, textile and fashion industry related start ups	Business developer
NGO	Fair action		Help develop companies environmental and social sustainability work	Project manager

## Footnotes

1 Not all initially selected cases agreed to participate in the research, and we therefore chose to omit one particular company with a renting business model from the analyzed sample.
