# Supplementary material for: Fashion Companies Pioneering with Eco-Innovations in the Swedish Fashion Industry: Motivations, Resources, and Cooperation
Source: Circ Econ Sustain. 2023 Jan 18:1–21. Online ahead of print. doi: 10.1007/s43615-022-00246-x (PMC9845818; doi:10.1007/s43615-022-00246-x)
Supplement: Supplementary file 1 — Supplementary file1 (PDF 859 KB) [file 43615_2022_246_MOESM1_ESM.pdf]

Research Protocol

Supplement to the paper "Fashion companies pioneering with eco-innovations in the Swedish fashion industry: motivations, resources and cooperation"

Authors: Naomi le Feber & Martijn Smit[1]

Date: 9 November 2022

[1] This protocol was based upon the original methodological appendix that accompanied the master thesis written by Le Feber under the guidance of dr. Smit at Utrecht University.

Contents

Contents ... 2
1. Methods ... 3
  1.1 Research population ... 3
  1.2 Research design, sampling method and data collection methods ... 3
  1.3 Observation units ... 3
  1.4 Selection method and sample ... 4
    1.4.1 Literature review ... 4
    1.4.2 Maximum variation cases ... 4
    1.4.3 Selection of critical cases based on location ... 6
    1.4.4 Final selection of critical cases ... 6
  1.5 Data collection ... 7
  1.6 Operationalisation ... 7
2. Ethics ... 8
3. Data analysis ... 9
  3.1 Transcription ... 9
  3.2 First phase of coding ... 9
  3.3 Second phase of coding ... 10
References ... 12
Appendix: code tree first phase of coding ... 13

1. Methods

1.1 Research population

Enterprises in the Swedish fashion and textile industry, that are implementing environmental innovations to increase sustainability and/or circularity of either their design, production and product choices or their entire business models, and other relevant actors that support eco-innovations, sustainability and circularity through the exchange of knowledge and other resources by cooperation. These are, based on the literature and fieldwork: suppliers, intermediate organisations (governmental and non-governmental) and research institutes.

1.2 Research design, sampling method and data collection methods

Explorative case study research, with information-oriented case selection. This sampling method maximises the utility of the acquired information from a small sample and requires selection of cases on the basis of expectations about their information content. The objective of the research was to acquire the greatest diversity of information and perspectives, for which selecting critical cases with maximum variation is the most appropriate (Flyvbjerg, 2006). Critical cases with maximum variation provide insight into fashion companies' reasons for eco-innovations, perceived limitations and coping mechanisms, and lastly cooperative behaviour to facilitate eco-innovations which might be different depending on size, geographical location, the niche market they operate in and/or the type of innovation(s) they implement. This has the added benefit of maximizing the usefulness of the acquired information from our relatively small sample.

Furthermore, the selected cases can also be considered as critical. This will provide information that permits a logical deduction (Flyvbjerg, 2006): if this is (not yet) valid for this case, then it can apply to all/no cases similar in size, geographic location and role. Therefore, both cases most likely and least likely to cooperate and cases most actively and least actively implementing environmental innovations in design, production and product were selected. This selection is based on the listed cooperation's and collaborations with other organisations and companies on their websites.

The sources of information used in this research were case interviews, conducted with representatives from fashion or textile enterprises, and representatives from research, industry, NGO's and governmental organisations. Interviews have been conducted with the (co-)founder or those who hold a position as project manager, sustainability manager, CSR and quality manager, product manager, production manager, head of public relations or are otherwise experts on the sustainability practices, innovations and collaborations of their company or organisation. These interviews were supplemented with extra documents provided directly by the respondents or by their websites.

1.3 Observation units

Fashion and textile enterprises, cooperative organisations initiated by the government, industry or research and innovation institutes.

1.4 Selection method and sample

In order to be able to select the most information rich cases several steps were taken.

1.4.1 Literature review

Firstly, relevant keywords were combined to create search terms within academic databases to identity the literature on sustainable business models, eco-innovations, and networks and collaborations within the general literature and literature on the fashion and textile industry. Searches were carried out through Web of Science, Google Scholar and Worldcat, using the following keywords: 1) sustainable business model, 2) circular business model, 3) sustainable innovations, 4) circular innovations, 5) cluster, 6) collaboration, 7) cooperation, 8) research, 9) networks, 10) industrial district, 11) CSR, and 12) sustainable strategies. These keywords were combined with: fashion industry, apparel industry, fashion company, textile industry, fashion design, fashion entrepreneurs or cultural industry to specify for the industrial context. Lastly, searches were carried out specifically for the Swedish context as well, adding: Sweden or Swedish to the search keywords.

Hereafter, governmental and industry specific sources were searched and consulted, to gather information on the most recent developments concerning the fashion industry. These sources included: the Report Mapping Sustainable Fashion Opportunities for SMES, published by the European Commission in September 2019, the report Is Apparel Manufacturing coming home? Nearshoring, automation, and sustainability – establishing a demand-focused apparel value chain, published by McKinsey Apparel, Fashion & Luxury Group in October 2018, the most recent Pulse of the Fashion Industry report, published by Global Fashion Agenda and the Boston Consulting Group (2018), and The State of Fashion 2019 report published by McKinsey & Company and Business of Fashion (2019).

1.4.2 Maximum variation cases

In order to choose maximum variation cases, the literature covering different sustainable and circular business models and innovations specifically in the fashion and textile industry was examined to make a categorisation. This categorisation included the relevant different actors involved in sustainable innovations in the fashion industry: intermediate organisations and platforms, fashion enterprises, fashion entrepreneurs, governmental agencies, research agencies, suppliers, sellers, trade unions. Furthermore, a categorisation of different sustainable and circular innovations and business models was made: innovations in design (including materials), production, product, service, or entire business model. Lastly, a categorisation based on company size was made, because the size of the company influences its innovativeness and ways of collaborating for innovative ideas and capacity (European Commission, 2019; Global Fashion Agenda & Boston Consulting Group, 2018; Weiblen & Chesbrough, 2015). These three categorisations provided a framework from which to search for and select cases in the Swedish fashion industry.

The majority of the European fashion industry consists of SMEs (European Commission, 2019). In Sweden, when looking at the total shares in numbers, 95% of the fashion enterprises are start-ups and micro-enterprises, 5% are SMEs and 0,1% a large company. However, considering their contributions to the total turnover shows a different situation: micro enterprises contribute 5% to total turnover, SMEs 44% and large

enterprises 30% (Sternö & Nielsén, 2015). Because the majority of turnover in the Swedish fashion industry comes from SMEs, the majority of cases selected are SMEs, complemented with start-ups and a large enterprises.

To select maximum variation and critical cases two methods have been used. The first method was searching for programs aimed at both research and industry partners to enhance sustainability and/or circularity in the Swedish fashion industry by means of cooperation and sharing of knowledge and experiences.

Two main players in the Swedish context were identified and consulted:

1. Mistra Future Fashion: This was a Swedish cross-disciplinary research program for circular fashion with research partners and industry partners. The program started in 2011 and was prolonged with a second phase from 2015 until 2019. Therefore, the enterprises which were cooperating in this program served as rich cases of information about being involved in cooperative organisations, researchers and other companies to search and implement environmental innovations.

   The companies approached through this method were: Uniforms for the Dedicated, Residues, Boob Design, Swedish Stockings, Houdini Sportswear and Filippa K. Furthermore, the board of the program was approached, by email and phone call, but they declined my request for an interview due to time limitations because they were busy closing off the program. Of the companies contacted through this method, many declined or did not answer the requests.

   The experts approached were: Mistra Future Fashion, Stockholm Fashion District, Swedish Fashion Council and Remake.

2. The national platform Textile & Fashion 2030. Textile & Fashion, initiated by the Swedish Government, is the national platform for sustainable fashion and textiles. The platform is running since the end of 2018 and a five-year assignment led by Smart Textiles, part of the Science Park Borås, in collaboration with the Swedish School of Textiles, The Swedish Fashion Council, The RISE Research institutes of Sweden, The Swedish Trade Federation, and TEKO, the Swedish trade and employers' association for companies working in the textile and fashion industry. The platform aims at putting Sweden at the forefront when it comes to development of a sustainable and circular fashion industry. The platform works actively with fashion enterprises, both micro, small, medium and large, by offering them access to knowledge (of sustainable and circular BM) and experience (with eco-innovations) through self-assessment, project support, seminars and workshops, training and the use of their tools and resources.

   The companies approached through this method were: T-Studio, No Mans Label and Rekotex, Gina Tricot. Experts approached through this method were: Borås ink, Textile & Fashion 2030 (project manager), Smart Textiles Borås (innovation leader and associate professor).

The second method employed to find representative cases was through web searches and websites analysis. Through google was searched for Swedish fashion brands, with headquarters in either Stockholm, Goteborg or Borås. Then the websites have been analysed to check whether and which environmental innovations (in design, production and/or service) had been implemented. This method allowed us to include companies who were not directly connected with the research programs or platforms, to give insight in the perspectives of companies outside these networks.

> The companies approached through this method were: Residues, As We Are Now, Rerobe, Sabine and Friends, Dedicated, Röhnisch, Nudie Jeans and Arket. One experts was approached through this method: Fair action.

1.4.3 Selection of critical cases based on location

Initially only companies in Stockholm were searched and asked to participate, because many fashion companies are located here. To enlarge the sample of critical cases, we expanded the coverage to companies located in Goteborg and Borås. Goteborg, because it hosts many headquarters of fashion companies and Borås, because it hosts the innovative Smart Textiles research program and incubator for innovative fashion tech start-ups.

1.4.4 Final selection of critical cases

Tables RP1 and RP2 below provide a systematic oversight of the companies and organisations contacted, their location, their environmental innovations. Only companies and experts that have accepted the request to participate in the research are included. All companies contacted have been included above.

Table RP1 Innovating companies

Size	Company	Location	Product:	Process:
Micro (1-9)	As We Are Now	Stockholm	X	X
	T-studio	Borås		X
	No Mans Label	Borås	X	X
Small (10-49)	Boob Design	Stockholm	X	
	Swedish Stockings	Stockholm	X	X
	Dedicated	Stockholm	X	
Medium (49-249)	Röhnisch	Stockholm	X	
	Nudie Jeans	Göteburg	X	X
Large (250+)	Arket	Stockholm	X	X

Table RP2 Approached experts

Type of organisation	Name and location	Involved parties and financiers	Activity	Interviewed
Industry	Stockholm Fashion District	Association of Trade Partners Sweden.	Business platform for fashion in Sweden.	Head of public relations.
Government	Textile & Fashion 2030	Smart Textiles; The Swedish school of	In collaboration with business and academia encouraging and coordinating	Project Manager

	- Borås	Textiles; Swedish Fashion Council; RISE; Teko; Svensk Handel	education, research, innovation with the aim of supporting environmentally friendly development in fashion and textiles.	
Research	Smart Textiles Borås	Science Park Borås; RISE; Swerea IVF; Incubator Borås; Vinnova, Västra Götaland region; Sjuhärads Kommunalförbund.	Engine of the Swedish textile industry since 2006 with over 500 research and business projects. It is an innovation environment in northern Europe for the fashion and textiles and textile technology.	Innovation leader
				Associate Professor/Responsible Smart Textile Technology Lab
Incubator	Borås Ink	Borås Stadshus AB	Personalised and customised business development for technology, textile and fashion industry related start ups	Business Developer
NGO	Fair action		Help develop companies environmental and social sustainability work	Project manager

1.5 Data collection

The interviews have been conducted between October 2019 and January 2020 through a phone call or face-to-face and have varying lengths between 40 to 90 minutes. The interviews were semi-structured and guided by a topic list.

The interviews conducted with the fashion enterprises included questions regarding their environmental innovations and what they perceived as challenges, necessary resources, supportive actors or organisations or and environments, and specific questions regarding collaborative interactions they had. The interviews with the organisations included questions about how they provided support or knowledge to companies through their activities, how they perceived the interest companies in their activities and how they supported collaboration and networking with the aim to improve sustainability in the industry.

1.6 Operationalisation

Previous research provided a multitude of factors influencing the implementation of environmental innovations by companies, both in general and in the context of the fashion industry. This multitude of factors has been organised in groups shown in the conceptual model in Figure RP1.

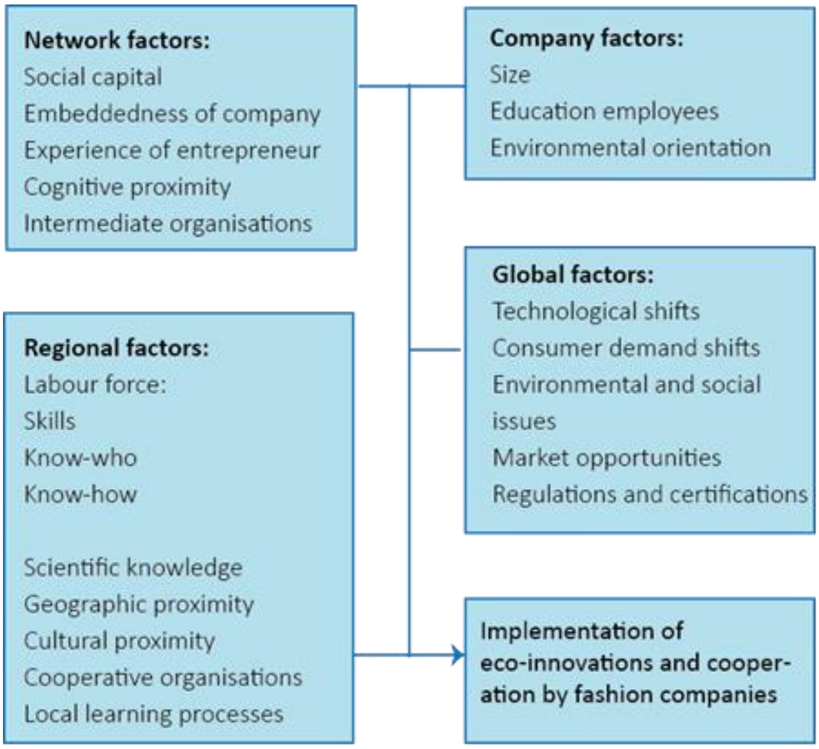

Figure RP1: conceptual model

2. Ethics

The research qualifies for informed consent, all participants have been personally approached and informed about the researcher, the research topic, the research questions, the benefits of participating and the end product. Furthermore, they have been fully informed about what participation involve: the questions I would ask, that it would be recorded, the time it would take and how I would use and care for their information. All the respondents have given their consent to be interviewed for my research by email and by phone, and to be recorded. They also gave consent for using the name of the company and when using a quote, they have been informed to give consent. The interview recordings are handled confidentially to secure privacy of respondents, only the researcher has access to the records and to the transcripts.

Sustainability and innovations can be sensitive topics to some companies. The research has taken into account that respondents cannot always share detailed information or personal views about particular environmental innovations, because these are secret/a competitive advantage. Therefore the focus of the interviews has been on the kind of innovations implemented, what motivates the company to innovate, what they perceived as beneficial for this implementation and elaboration in the cooperative relations listed on their websites. Furthermore, profitability and sustainability together are a sensitive combination. Companies might be hesitant to talk about how environmental innovations have to be profitable or economically viable. Therefore, in the interviews respondents have not been directly asked about profitability. It was often naturally raised by the respondents as a topic, and then respondents were asked to elaborate if necessary.

3. Data analysis

Prior knowledge: The examined literature has provided a framework with sensitizing concepts to start analysis with. These have a broad and general description, and have been used to create order and clarification in the collected data. A schematic overview of the sensitizing concepts is shown in Figure RP2.

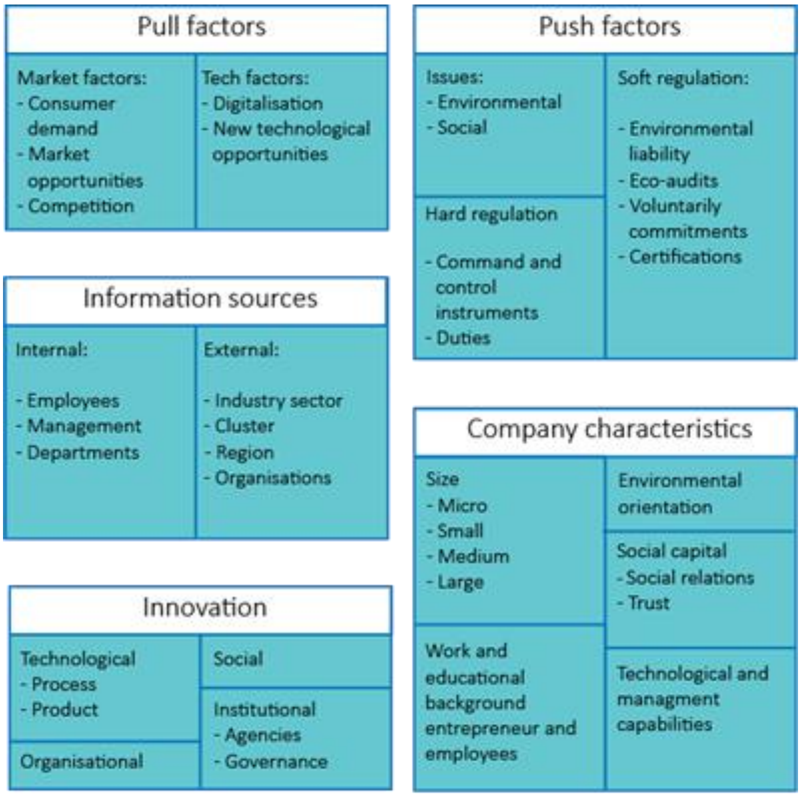

Figure RP1: Sensitizing concepts

3.1 Transcription

Fourteen of the fifteen interviews have been fully transcribed, one walk-along tour and interview has been processed as field report. These transcripts and the field report have been added to the Nvivo 12 software. Frequent pauses, distracting use of fillers to search for expressions and unfamiliar/unintelligible words have not been transcribed, because these were not relevant for the answering of the research questions.

3.2 First phase of coding

In the first phase of coding each interview was read thoroughly and codes were allocated to the transcripts guided by the sensitizing concepts. Therefore, the analysis started off in a deductive manner. Coding guided by sensitizing concepts resulted in too many and too detailed codes. The sensitizing concepts provided a framework to start with, but the concepts were not specific enough and harboured a large number of very diverse fragments after coding the first set of interviews. Therefore it was combined with in vivo and open coding. This meant the text was examined in more detail, searched for fragments that contained more detailed information or new information. These were organised under the sensitizing concepts or under a new concept, when the information was new.

Both in vivo codes were used, whereby words or formulations were derived directly from the text, and summarizing codes, whereby the full answer of a respondent has been summarized this in one code. It was not always possible to code in vivo, because some respondents used long explanations or stories to answer the questions.

When coding the last transcripts of the companies no new codes were found: the information of these cases could be separated in fragments that could be covered by already existing codes. Thereby reaching a point of saturation.

After coding the companies transcripts, the transcripts of experts were coded, following the same procedure. Furthermore, these organisations became concepts themselves, with codes with fragments about their relevance, activities, target groups etc. These respondents were not part of the research population, but provided information about the research population, the whole fashion industry and/or environmental innovations.

Before finalising the first phase of coding, all the codes and associated fragments were examined and some fragments were recoded to a better fitting code. The end result was a very long and detailed code tree, which as been added as an appendix.

3.3 Second phase of coding

In the second phase of coding, firstly every code was checked thoroughly for the use of synonyms and relevance for answering the research questions. Thereafter a round of axial coding was carried out. This is a more abstract process and consists of coding around several single categories. The data was put back together in new ways after the open coding, by connecting categories or merging certain categories in one. The codes have been categorised based on their relevance for answering the research questions.

RQ1 What is the relationship between the pioneering fashion companies' size and their ability and willingness to develop and implement (product and/or process) eco-innovations?

Categories

- Enterprise characteristics, eco-innovation, sector-cooperaration

Sub categories

- Company differences
- Ability to acquire/have access to knowledge and inspiration sources
- What they need/necessary factors
- What they do: process, product or BM innovation
- With whom companies cooperate, their motives and what kinds of cooperation can be distinguished.

RQ2 Why are pioneering fashion companies' developing and implementing eco-innovations?

- pull factors
- push factors,
- enterprise characteristics
- subcategories: environmental motivation, internal driver, perception of sustainability

RQ3 What do pioneering fashion companies perceive as limiting factors to their ability to develop and implement eco-innovations and how do they cope with these?

Categories

- enterprise characteristics, eco-innovation, sector cooperation, NGO Fair Action, Incubator's role, Investor interest

Sub category

- What limitations they face
- Ability to acquire/have access to knowledge and inspiration

RQ4 What are the motivations of individual actors to cooperate horizontally or vertically in the fashion industry in order to search, develop and implement eco-innovations?

Categories

- sector cooperation
- enterprise characteristics:
  - subcategories: network connections, business culture
- eco initiatives:
  - subcategory: necessary factors - cooperation listed as necessary?

References

European Commission. (2019). *Support Report Mapping Sustainable Fashion Opportunities for SMEs*.

Flyvbjerg, B. (2006). Five Misunderstandings About Case-Study Research. *Qualitative Inquiry*, *12*(2), 219–245. https://doi.org/10.1177/1077800405284363

Global Fashion Agenda & Boston Consulting Group. (2018). *The Pulse of the Fashion Industry 2018*. https://globalfashionagenda.org/product/pulse-of-the-fashion-industry-2018/

McKinsey & Business of Fashion. (2019). *The State of Fashion 2019: A Year of Awakening*. https://www.mckinsey.com/~/media/mckinsey/industries/retail/our%20insights/ten%20trends%20for%20the%20fashion%20industry%20to%20watch%20in%202019/the-state-of-fashion-2019-final.pdf

Weiblen, T., & Chesbrough, H. W. (2015). Engaging with startups to enhance corporate innovation. *California Management Review*, *57*(2), 66–90. https://doi.org/10.1525/cmr.2015.57.2.66

Appendix: code tree first phase of coding

Eco Initiatives

- Global Initiatives
- National Initiatives
  - Interest for it
  - Internal structure
    - Neutrality
    - Stimulates interorganisational cooperation
    - Test Ideas & feedback
  - Physical place
  - Target group
  - What they do
    - Activities
      - Projects
      - Seminars
      - Workshops
    - Bring stakeholders together
    - Challenge
    - Gather latest information
    - Inspire
    - Tailored support
  - Who they are
  - Why Founded
- Local Initiatives
  - Science Park's Do Tank
    - Asking the right questions
    - Drive Innovation
    - Encouraging projects
    - Linking critical stakeholders
    - Providing circular technologies
    - Transfer knowledge
    - How they attract attention
      - Publicity
      - Talks

Eco-innovation

- BM innovation
- Companies differ
- Knowledge sources
  - Accessibility
  - External
    - Global VC & Start-up generator
    - Cluster
      - SFD
    - Industry sector
      - Kemikaligruppe
      - STICA (Swedish Textile Initiative for Climate Action)
      - STWI (Sweden Textile Water Initiative)
      - Suppliers
    - Region

                - Reports
                - Research and education
                    - Boras Univeristy
                    - Textile & Fashion 2030
                - Tech sector – related variety
            - Importance
            - Internal
            - Proximity
        - Limitations
            - Fast fashion
            - Fixed routines and optimisation
            - Lack of certain knowledge
            - Lack of demand
            - Lack of money
            - Lack of qualified staff
            - Many sustainability standards
            - Need for profit
            - Too small volumes
        - Necessary factors
            - Accessible info
            - Applicability
            - Ask feedback
            - Be relevant solution for demand
            - Competitive advantage
            - Continually learning
            - Cooperation and transparency
                - Know who to cooperate with
            - Creativity
            - Digitisation
            - Don't start small
            - Educating consumers
            - External pressure and support
            - Fast decision making process
            - Figure out demand
            - Financial resources
            - Increased demand
            - Interest for sustainability
            - Keep up to date
            - Machinery
            - New BM
            - New routines
            - Personnel
            - Reach out
            - Search info
            - See profitability
            - Start small
            - Strategic systematic change
            - Suppliers
            - Take initiative
            - Time
            - Whole company on board

        - Willingness
        - Working groups
    - Necessary knowledge
    - Process
        - Input raw materials
        - Production process
    - Product
        - Exchanging product components
        - Integration new product component
        - Optimisation product components
        - Reclaiming and recycling
    - Starts internally
    - Takes time
    - Technological
    - Transparency
- Enterprise characteristics
    - Business culture
        - Vision
    - Capabilities
    - Education
    - Environmental motivation
    - Growth
    - Industry background
    - Internal driver
    - Leading role
    - Network connections
    - Perception of sustainability
    - Size
    - Taking risks and being bold
- Incubator's role
    - Accessibility
    - Connecting and building networks
    - Lower cost of marketing innovation
        - Convince investors
        - Funding
        - Privileges
    - Start-ups change in focus
    - Support start-ups
    - Tech and textile focus
- Investor interest
    - Patents
- NGO Fair Action
    - Communication with companies
    - Problem of reachability
    - Research
    - Supervisor of system
    - Target companies
        - Why
        - Their focus area
            - Living wage corporate responsibility
            - RECENT transparency
- Personal thoughts

Pull factor

- Competition
- Consumer demand and awareness
- Market opportunities
- Path dependency
- Tech opportunity

Push factor

- Environmental challenges
- NGO pressure
- Overproduction
- Regulation
  - Hard
  - Soft
- Transparency

Role of place

- Access to customers
- Access to machinery and labs
- Being of the radar
- Closeness to industry and workforce
- Connecting and sharing
- For knowledge transfer
- Innovative and supportive atmosphere
- Personal motives
- Sustainable country

Sector cooperation

- Cooperate with likeminded
- Stockholm Fashion District
  - Educative
  - Linking actors
  - Place to connect
  - Supported by government
  - Supports innovation through collaboration
  - Sustainability support
- Transparency
  - Sharing suppliers
- Why important
  - Inspire others
  - See what others are doing
  - Staying up to date
- With organisations
- With other businesses
- With suppliers

Small country
